# Knowledge Engine, Wisdom System: Aligning Generative AI With the Relational and Epistemic Foundations of Primary Care

**DOI:** 10.7759/cureus.111382

**Published:** 2026-06-23

**Authors:** Joachim P Sturmberg, Carmel M Martin

**Affiliations:** 1 General Practice, University of Newcastle, Newcastle, AUS; 2 General Practice, Monash University, Brisbane, AUS

**Keywords:** ai governance, artificial intelligence, clinical decision-making, clinical epistemology, continuity of care, fast and slow learning, general practice, large language models, primary care, socio-technical integration

## Abstract

Many patients now turn to generative AI for health advice before seeing a doctor. However, there is little evidence about how well it works in primary care. Research focused on general practice is limited, and real-world harms are often missed because there is no standard way to record AI-related incidents. The gap between what AI provides and what general practice needs is not just technical; it is structural and requires active oversight. AI can recognise patterns, summarise documents, and organise data, but it does not have the judgement, long-term patient knowledge, or moral reasoning that define good general practice. Evidence shows that careful use of AI can help, but careless use can cause real harm. To safely use AI, we must protect what makes primary care effective: knowing patients over time, reasoning through diagnoses across visits, and building healing relationships that combine clinical skill with whole-person care. No training set or workforce change can replace this. Evaluation should look beyond accuracy, considering continuity of care, the risk of losing clinical skills, patient experience, and fairness. General practitioners (GPs) must take an active role in guiding how AI is used, rather than simply adding it to current practice.

## Editorial

Introduction

Many patients now use generative AI as their first stop when they have a health concern. Lutes and Hughes call this shift the “front door” of health care, moving from the doctor’s office to an algorithm [1]. These AI tools often sound smooth and comforting, but sounding confident does not mean they truly understand or provide safe advice. They work by matching patterns in data, not by reasoning or drawing on real-life experience [2-7].

These concerns are sharper in primary care, where clinicians deal with longitudinal patient relationships, ethical choices, and context-aware decisions that are among the most demanding in medicine [8-10]. Nowhere is this clearer than in the management of diagnostic uncertainty. Up to 35% of patients come in with unexplained or undifferentiated symptoms [11]. A diagnosis typically emerges across multiple visits, making time itself a key diagnostic tool in primary care [11].

Current AI systems cannot match these abilities. Managing uncertainty in primary care depends on building meaning from repeated encounters with the same patient over time. AI has no equivalent of that accumulated, relationship-based knowledge [7,12].

Still, we have limited evidence about the benefits and risks of AI, so findings should be interpreted carefully. Research on AI in primary care is just beginning. Most studies look at specific technical tasks like diagnosis support, chronic disease management, or documentation. Few examine how AI affects relationships, ongoing clinical thinking, or patient trust [13,14]. Real-world harms are also rarely reported, since there is no standard way to track AI-related incidents in general practice [15].

**Figure 1:**
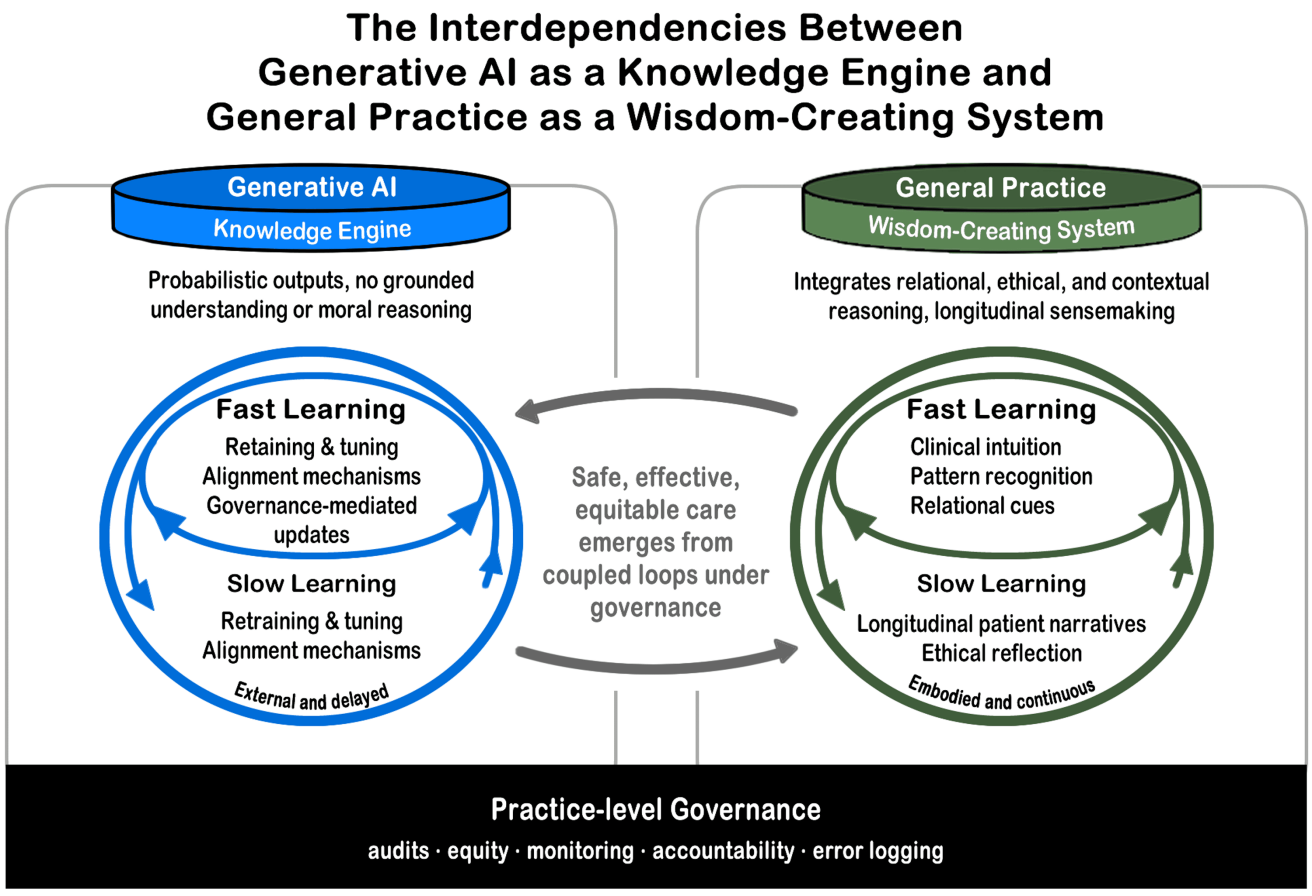
Generative AI as Knowledge Engine and General Practice as Wisdom-Creating System Generative AI works as a knowledge engine, giving probabilistic answers using processes similar to fast learning (like real-time analysis and summarising) and slow learning (such as retraining and updating). However, it does not have real understanding or moral judgment. General practice, on the other hand, creates wisdom by combining quick clinical intuition with slow, ongoing sensemaking, ethical decisions, and relationship-based care. These human processes are continuous and adapt to context in ways AI cannot match. Safe and fair results come from connecting these learning processes under proper practice-level oversight. Author-generated image created using Adobe Fireworks (Adobe Systems, San Jose, CA, USA).

This paper aims to fill that gap. It looks at not only what AI can do, but also when it can be used safely and what happens when it is not. We focus on three main questions: What can these systems really achieve? What are their limits? And how should primary care manage AI integration? This paper describes the state of AI integration in primary care as of early 2026. Several capabilities characterised here as emergent - including AI-assisted prescribing and longitudinal pattern detection - are now entering formal evaluation [16]; the governance and epistemological questions it raises apply with equal force to those developments. Figure 1 shows our main idea: AI acts as a knowledge engine, while general practice creates wisdom. Safe integration depends on learning from both and having proper oversight [13,17]. Instead of just asking if AI can help, we ask what makes its use safe, fair, and true to the core values of primary care [8,9,17].

Table 1 illustrates where the knowledge engine meets the wisdom system in a complex consultation. Table 2 maps analogous but not equivalent fast and slow processes in AI and general practice.

**Table 1:** When the Knowledge Engine Meets the Wisdom System: AI and GP Roles in a Complex Consultation A 68-year-old woman with diabetes and chronic obstructive pulmonary disease attends her general practitioner (GP) following a recent emergency department visit. eGFR: estimated glomerular filtration rate; FEV1: forced expiratory volume in the first second

Useful assistance …		… Requiring improvements
The AI system accurately summarises medication changes and key discharge instructions, highlighting the steroid taper, addition of a new inhaler, and the need for follow‑up.		
Where problems emerge	→	Ideal integrated function
When prompted for a "suggested management plan”, the system confidently recommends increasing metformin, initiating smoking cessation counselling, and switching inhalers. These suggestions appear plausible but fail contextually:		An effective AI collaborator would:
It assumes she smokes (a training data bias)- she never has.		Cross‑check discharge summary against structured patient data, flagging inconsistencies (e.g., "New metformin dose conflicts with documented eGFR 52 and prior intolerance").
It overlooks her borderline renal function and previous metformin intolerance.		Medication reconciliation: Compare current orders against allergies, renal function, and drug interactions, highlighting unresolved discrepancies.
It fails to recognise that she already demonstrates excellent inhaler technique.		Trajectory visualisation: Plot HbA1c, eGFR, and FEV1 trends over 24 months with automated annotations for exacerbations and interventions.
These errors are presented with confidence, without signalling uncertainty. When asked if the advice is “safe”, the system responds with legalistic disclaimers - products of alignment rules rather than genuine reassurance.		Care gap detection: Flag overdue pneumococcal vaccination and annual eye screening per jurisdictional schedules for her age/comorbidities.
		Equity audit: Note "This regimen shows 20% lower adherence in non-English speakers per local data – consider simplified instructions".
		Uncertainty quantification: Rate recommendations by data completeness (e.g., "Low confidence: no recent weight/smoking status documented").
Human judgment prevails		
Through dialogue, the GP uncovers bereavement stress, caregiving responsibilities, and transport barriers – factors beyond the AI’s inferential reach. Together, they co‑create a feasible plan that maintains metformin, addresses grief, and accommodates her circumstances.		
Learning from failure		
The practice logs the AI’s incorrect assumptions as part of its governance and implementation learning cycle.		
Key lesson		
AI excels as a data librarian, anomaly detector, and care gap identifier – easing cognitive load while deferring contextual reasoning, ethical judgment, and relationship-building to clinicians.		

**Table 2:** Analogous but Not Equivalent: AI and General Practice Cognitive Modes Compared Across Fast Thinking, Slow Reasoning, Longitudinal Sensemaking, and Ethical Judgment * Ideal integrated tasks are outlined in Table 1.

Cognitive mode	General practitioner capabilities	Generative AI capabilities* (current and emergent)	Safety and bias considerations	Implications for evaluation
Fast thinking (intuitive, real-time)	General practitioners use pattern recognition grounded in lived clinical experience, relational continuity, and situational awareness developed over time.	Current systems support triage, risk flagging, and text summarisation. Emerging systems integrate real-time data streams, large language model–based probabilistic outputs, and sensor-enabled alerts.	There is a risk of automation bias, whereby confident but context-poor recommendations may be over-trusted. Algorithms may also reproduce historical or population-level inequities.	Evaluation should examine effects on decision framing, clinician reliance, false reassurance, and differential impacts across patient groups.
Slow thinking (reflective, deliberative)	General practitioners integrate longitudinal clinical history, patient goals, ethical reasoning, and uncertainty management in reflective decision-making.	Current tools implement guideline engines and decision trees. Emerging systems aim to synthesise multimodal records, generate goal-aligned recommendations, and simulate shared decision support.	Uncertainty may be obscured, and medical-industrial priorities embedded. There is a risk of deskilling reflective clinical reasoning if outputs are treated as authoritative.	Evaluation should assess influence on professional judgment, continuity of care, and alignment with patient values and preferences.
Longitudinal sensemaking	General practitioners interpret patient trajectories over time, anticipate future needs, and maintain narrative coherence across episodes of care.	Current AI systems are largely episodic and task-based. Emerging approaches attempt longitudinal pattern detection and predictive modelling across datasets.	Contextual meaning may be lost and care fragmented if longitudinal outputs are not integrated with relational and care network knowledge.	Evaluation should focus on impacts on continuity, coordination, anticipatory care, and patient and their care network trust over time.
Ethical and narrative framing	General practitioners define what matters to patients, exercise moral judgment, and steward care within family and community contexts.	Generative AI systems do not possess normative reasoning and infer values only indirectly from data. Emerging multi-agent deliberation frameworks (e.g., CogniAlign [18]) operationalise moral judgment as an emergent property of structured inter-agent debate, grounding outputs in disciplinary argument rather than single-model alignment.	Goals may be misaligned, and value assumptions remain unexamined, with risks of inequitable prioritisation.	Evaluation should examine governance arrangements, accountability, and mechanisms for human oversight of value-laden decisions.

What generative AI can do

Large language models (LLMs) generate text by finding patterns in large datasets and picking likely next words. They do not use experience, understand cause and effect, or truly grasp meaning [5-7,19]. Treating their outputs as if they are expert clinical judgments is a basic error because it confuses *sounding right* with actually *being right* [5,6]. These systems do not know patients, cannot consider values, and have no way to set clinical priorities [8].

Scale makes this worse. When models train on their own outputs, performance degrades, variety narrows, and existing biases grow [20]. The larger and more opaque the model, the harder it becomes to align with professional and ethical norms [3,4,19-21].

In primary care specifically, the evidence is troubling. Across 21 LLMs tested on standard clinical scenarios, more than 80% of differential diagnoses were wrong at early patient presentation, exactly when patients are most likely to rely on AI [7]. AI developers themselves admit that safeguards such as alignment tuning and human feedback loops remain partial and unreliable [6,20].

Three implications for primary care follow: AI outputs should be treated as provisional artifacts requiring clinical interpretation, not as autonomous judgments; Biases and blind spots, especially those affecting underrepresented populations, must be anticipated rather than discovered in retrospect; Apparently, ‘safe’ behaviour often reflects training limits or prompt engineering rather than genuine understanding. Clinicians should not be reassured by cautious-sounding AI responses; they may fail without warning in unfamiliar clinical situations.

A pragmatic stance for primary care

Despite these limits, generative AI offers real value for well-bounded tasks: summarising documents, drafting letters, and supporting coding [3,4]. The evidence supports this. Clinicians with LLM decision support made 16% fewer diagnostic errors and 13% fewer treatment errors across 39,849 primary care visits [22]. An LLM co-pilot increased the detection of serious prescribing errors by 1.5-fold compared with pharmacists alone [23]. A randomised trial found that ambient AI scribes reduced documentation time and clinician burnout across 238 outpatient physicians [24]. AI can take over administrative tasks, allowing clinicians to focus on their patients [25]. And AI’s capacity for handling large amounts of data, maintaining longitudinal records, detecting risks at the population scale, is a genuine and underacknowledged strength [26,27].

However, the evidence of harm is equally concrete. Whisper, deployed by over 30,000 clinicians despite OpenAI’s own warnings against use in high-risk domains, invented words in approximately 1% of recordings; 38-40% of those were rated harmful or concerning, including fabricated medications and offensive language inserted into clinical records. At least one platform erased the original audio, making checking impossible [28]. Harm takes subtler forms, too. A patient who consulted ChatGPT about symptoms after cardiac ablation received reassuring but incomplete responses, delaying his transient ischemic attack (TIA) diagnosis [29]. A study of over 20,000 primary care visits found ambient scribe use linked to a lower likelihood of a depression-related intervention despite greater symptom documentation, a reminder that what gets recorded and what gets acted on are not the same thing [30]. This kind of misalignment is emblematic of emerging AI-related risks, with ECRI naming AI chatbot misuse the number one health technology hazard for 2026 [31].

Experimental frameworks such as CogniAlign [18] aim to put moral judgment into practice through structured debate among AI agents. These are promising, but do not fix AI’s deeper limits: no real understanding, no ongoing memory, and fragile handling of uncertainty.

The best way to think about AI is as a fast, junior assistant. It is good at recognising patterns and generating text, but it cannot set goals, understand values, or take responsibility. Its usefulness depends completely on careful human oversight [10,32].

What general practice uniquely provides, and what uncritical AI integration can erode

It is important to know both the strengths and limits of AI, but that is only part of the picture. We also need to recognise what general practice offers that cannot be replaced, and what might be lost if AI is not used carefully.

The doctor-patient relationship is the moral and clinical centre of medicine. It is the foundation from which healing proceeds, and the most defining characteristic of general practice [8,33,34]. A GP who has known a patient for a decade holds something that cannot be easily replicated. They know how that person shows distress, what they share and what they hold back, how family life shapes their health choices. They understand how social factors such as housing insecurity, bereavement, family stress, and language barriers affect how much a patient can engage with care. And they know what matters to that patient when a hard decision has to be made. That same care may also notice a suspicious skin lesion the patient has not mentioned, and that no AI input would have surfaced, a reminder that whole-person care covers what is seen as much as what is said. It is this sustained, evolving relationship, built through repeated encounters, shared history, and careful attention across a lifespan, that makes general practice a *healing practice* rather than a *transaction*. These are exactly the abilities that automation cannot displace: ongoing continuity across time, diagnostic curiosity under uncertainty, and the relational and moral dimensions of care [35].

These abilities rest on deep clinical knowledge and training, the products of years of medical education, clinical experience, and pattern recognition built through sustained, supervised practice [7,8,36,37].

This kind of tacit knowledge [38], built around each person’s unique situation rather than general data about populations [8], is something AI cannot develop. AI systems collect and process data, but they do not capture the deeper meanings that come from relationships. These meanings often appear in conversations over many years. The main point is clear: when AI systems skip or shorten the relationship between doctor and patient, they risk turning meaningful care into simple data processing and missing key information that makes primary care work. Table 1 shows this clearly: the general practitioner (GP) found out about bereavement, caregiving, family stress, and transport issues through conversation, things no AI could have detected.

The difference between what AI provides and what general practice needs is not just technical; it is a structural issue that requires oversight. Protecting the time and conditions needed for real clinical and relationship-based care is essential. We should not let efficiency in paperwork become a reason for shorter visits or weaker relationships that distance clinicians from patients [13,14,25]. There are five more areas of risk that need attention:

First, automation bias; clinicians defer to confident-sounding AI recommendations even when those recommendations are wrong. The management changes proposed by the AI in that same consultation were unsafe for this particular patient, yet were presented without any signal of uncertainty [39]. Without active human scrutiny, such errors spread undetected.

Second, algorithmic bias; training data that under-represent populations by age, ethnicity, language, or other conditions produce outputs that reflect and deepen those gaps. Published evidence shows that AI chatbots give different clinical recommendations for identical cases depending on patient race, ethnicity, gender, and financial status [21,40]. A system that performs well on average may perform worse for the patients most in need.

Third, accountability erosion; when clinical decisions are shaped by AI outputs, the lines of professional responsibility blur. If a clinician follows an AI recommendation that harms a patient, legal and ethical accountability remains unresolved, underscoring the need for clear frameworks across clinical, legal, and regulatory domains [40].

Fourth, data privacy and security; patient data processed by commercial AI systems creates obligations that clinicians must understand. Where is it stored? Who can access it? Could it be used to retrain the model? [14,20,40].

Fifth, and most insidiously, deskilling, addressed in the following section, is the risk that cuts deepest into the long-term strength of the profession.

Towards integration: fast and slow learning as a governance framework

Generative AI is quick and skilled at recognising patterns, sorting data, and making probability-based predictions. However, it does not have deep understanding, long-term memory of patients, or moral judgment, qualities that are central to general practice. To safely combine AI and general practice, we need a clear framework. Kahneman’s idea [41] of fast, automatic thinking (System 1) and slow, deliberate thinking (System 2) helps us compare the two. This does not mean AI and clinical reasoning are the same, but it shows where their fast and slow processes are alike, where they differ, and where oversight is needed. Table 2 shows how these similar but not identical processes fit into the four main thinking modes of clinical practice.

The map reveals both the potential and the limits of integration. AI fast processing operates in real time: pattern recognition, signal detection, and summarising structured and unstructured data. AI slow processing occurs through periodic retraining, alignment updates, and externally managed improvement; unlike clinical slow learning, it is stop-start and happens outside the system itself. GP fast learning draws on clinical training and knowledge that builds into intuition honed by direct experience, relational continuity, and situational pattern recognition [36]. GP slow learning includes ethical reflection, narrative integration, and system-level awareness, processes that are embodied, continuous, and context-sensitive in ways AI slow processing is not.

Integration is possible, but it does not happen automatically. It requires a deliberate effort to connect these learning processes. AI outputs are used in clinical care, and any failures are identified and reviewed. This feedback helps improve both the technology and professional skills [17,42]. This is not just a technical setup. It is governance. Institutions must ensure that human judgment stays active, critical, and central.

Deskilling: the central threat to integration

Deskilling grows gradually and silently. When clinicians treat AI outputs as final answers rather than suggestions, the habits of independent clinical reasoning, tolerance for uncertainty, reflective judgment, and comfort with complexity begin to atrophy [13,14].

Over time, the ability for independent clinical thinking may weaken, along with tolerance for diagnostic uncertainty and confidence in handling ethically complex cases without algorithmic support. A clinician whose reflective ability has faded is less able to catch AI errors, less able to exercise independent judgment in novel situations, and less able to speak up for patients whose needs fall outside the algorithm’s training. This is not an individual failure. It is a systemic risk: unaddressed deskilling quietly degrades the quality of, and ultimately trust in, primary care [13,14].

Aviation offers an instructive parallel. When autopilot became standard, pilots who over-relied on automation showed worse performance precisely when it mattered most, at the moment the system failed, and skilled flying became essential [39,43]. Primary care faces a similar challenge. AI is least reliable in situations that most demand clinical wisdom, undifferentiated presentations, diagnostic uncertainty, ethically complex decisions, and the deeply personal dimensions of illness and care that no training set can capture. These are the cases where deskilling poses the greatest danger.

Governance frameworks need to do more than just track how AI performs. They must actively protect the environment needed for clinical learning. This means making sure that careful, independent clinical reasoning is a core part of training and supervision, not just an extra. It also means making sure that pressure to see more patients does not push out time for reflection. Professional development should focus on building the skills that are most at risk from AI use [10,32,42]. Without these protections, *AI integration is substitution by stealth*.

Medical education must respond to this challenge with urgency. Primary care doctors need a strong enough knowledge and experience base to spot when AI is likely to be wrong. That requires strengthening rather than cutting the foundational training in clinical reasoning, human biology, and the limits of the evidence base on which AI systems are built. Clinicians also need continual, required training in the specific strengths and failure modes of the AI tools they use in practice. Understanding how scientific knowledge is generated matters here. Evidence-based medicine shapes the information systems that train AI. Where the source literature has blind spots, the AI carries them too. This is not a technical elective; it is a core clinical skill for the AI era [35].

This issue goes beyond individual doctors. Someone who is good with people but lacks clinical training, even when paired with an AI system, cannot do what a GP does. Relationship skills and clinical skills are not separate; they cannot be divided between different people or systems. The wisdom of general practice comes from applying clinical knowledge to individuals over time. If we split relationship-building from clinical care, giving one part to a navigator and the other to an algorithm, we lose the integration that makes primary care safe and effective. This also removes the key skill needed for AI integration: knowing when the AI is wrong, using clinical judgment in context, and taking responsibility for patient outcomes [36,41].

Patient perspectives: the human test of integration

The success of AI integration cannot be judged solely by technical metrics. The real test is whether it holds from the patient’s perspective: whether the gains AI delivers feel like improvements in care, and whether what makes general practice valuable is preserved rather than lost.

The evidence here is cautionary [44,45]. Many patients value AI’s ease of access, such as health information outside clinic hours, rapid answers to straightforward questions, and a low-threshold contact point when waiting times are long. But patients also say clearly that they do not want AI to replace their relationship with their GP. Trust, continuity, and the sense of being known as a person, not a case, remain central to what patients value in primary care [43,44]. Research shows that concerns about privacy, accountability, and loss of human connection increase sharply when patients learn AI may be involved in clinical decision-making rather than administrative tasks [44,45].

Equity deepens these concerns. Patients from diverse language backgrounds, those with lower digital literacy, and those with limited technology access may be simultaneously most reliant on AI as a first contact and least able to critically evaluate its outputs [31]. If AI becomes the primary gateway to health information, its quality must be judged against the needs of those with the fewest alternatives, the patients for whom the stakes of getting it wrong are highest.

These considerations point to a form of oversight the technical literature rarely names - relational governance [46], governing through trust, commitment, and sustained relationships rather than rules and metrics alone. Applied here, it means active, ongoing attention to whether AI integration strengthens or weakens the trust, continuity, and equity that define good primary care from the patient’s perspective [44,45]. Without it, what is gained in efficiency risks being lost in humanity.

Future roles of GPs in an AI-enabled system and implications for evaluation and governance

The preceding sections set out what is at risk. This and the next section address what primary care should do about it. As AI takes on data-heavy tasks, GPs remain essential for ongoing clinical thinking, relational care, and ethical judgment. These skills cannot be replaced. They rest on deep clinical knowledge that no algorithm and no relationally skilled but clinically untrained practitioner can provide [8-10,35]. Yet these are the very skills that decades of volume-based training and incentive structures have slowly crowded out. This makes the profession more exposed to AI substitution at exactly the moment when that exposure matters most. Each of the risks identified, automation bias, unfair algorithms, gaps in accountability, and deskilling, points to a bigger, not smaller, role for GPs.

As trajectory stewards, GPs guide the course of patient care across time and changing circumstances, integrating clinical data with life context. As record editors and curators, they ensure AI-generated documentation accurately reflects nuanced clinical reality, and guard against the hallucination and omission errors now documented in deployed systems. As system teachers and learning partners, they refine AI performance through feedback, annotation, and governance participation in everyday clinical settings. As patient advocates, they prioritise individual values, equity, and trust over algorithmic defaults, especially for patients most exposed to AI's failure modes. And as research and governance contributors, they shape AI evaluation, safety protocols, and implementation within learning health systems, while advocating for the GP-specific evidence base that currently does not exist.

Technical performance metrics such as accuracy, precision, and recall are necessary but not enough. The evidence surveyed shows that a system can perform well on benchmarks yet cause harm in real-world primary care contexts; the ambient scribe that documented symptoms without triggering intervention; the transcription tool that invented words in clinical records; the chatbot that delayed a TIA presentation. Evaluation frameworks must therefore look beyond technical outputs to assess six further dimensions [10,13,15,32]:

Continuity of care asks whether AI-mediated efficiency is displacing relational time, and what effects this has on longitudinal relationships and trajectory stewardship. Professional judgment and ethical reasoning ask whether human oversight is being preserved in value-laden decisions, and whether active resistance to automation bias is being built into clinical practice. Equity and patient trust ask whether the system performs differently across demographic groups, and whether the relational conditions patients value most are being maintained. Deskilling asks whether reflective clinical reasoning is being monitored for atrophy, and whether governance mechanisms are actively protecting the conditions for clinical learning. Patient experience asks whether patients feel known, respected, and appropriately involved in decisions, regardless of how much AI is being used in their care. And harm reporting asks whether standardised AI incident-reporting infrastructure is being built in primary care, equivalent to the systems that already exist for medication errors and adverse events.

Conclusion

Generative AI acts as a knowledge engine, but it is not a clinical mind. General practice is a system for creating wisdom, not just processing information. The difference between them is not a problem to solve; it is the space where clinical judgment, relationships, and ethical reasoning happen, and it needs careful oversight rather than elimination. The published evidence now supports both sides of this argument. Carefully governed AI reduces errors and documentation burden. Carelessly deployed AI invents words in clinical records, gives biased advice along demographic lines, and delays time-critical presentations. What determines which outcome prevails is governance. The absence of a standard harm-reporting system for primary care AI means the conditions for good governance are currently invisible to the systems responsible for creating them.

The risks of uncritical integration are documented, cumulative, and fall hardest on those already disadvantaged, for whom AI may be simultaneously most accessible and least safe. When used well, AI frees GPs to focus on what only they can do: building long-term relationships with patients, using moral judgment, and blending clinical evidence with the human side of illness and care. If used carelessly, AI can increase bias, weaken clinical judgment, and disrupt care for those who need it most. The goal is not just to adopt AI, but to ensure it supports rather than replaces the wisdom that defines good primary care, and to create governance systems that make these conditions clear, trackable, and enforceable.
